# How Exposures to Biologics Influence the Induction and Incidence of Asthma

**DOI:** 10.1289/ehp.8379

**Published:** 2006-01-26

**Authors:** Darryl C. Zeldin, Peyton Eggleston, Martin Chapman, Giovanni Piedimonte, Harard Renz, David Peden

**Affiliations:** 1 Division of Intramural Research, National Institute of Environmental Health Sciences, National Institutes of Health, Department of Health and Human Services, Research Triangle Park, North Carolina, USA; 2 Department of Pediatrics, Johns Hopkins University, Baltimore, Maryland, USA; 3 Department of Medicine, University of Virginia, Charlottesville, Virginia, USA; 4 Department of Pediatrics, West Virginia University, Morgantown, West Virginia, USA; 5 Department of Clinical Chemistry, University of Marburg, Marburg, Germany; 6 Department of Pediatrics, University of North Carolina, Chapel Hill, North Carolina, USA

**Keywords:** asthma, allergy, allergens, endotoxin, respiratory virus, immunoglobulins, tolerance, leukotrienes, neurotrophins

## Abstract

A number of environmental factors can affect the development and severity of allergy and asthma; however, it can be argued that the most significant inhaled agents that modulate the development of these conditions are biologics. Sensitization to environmental allergens is an important risk factor for the development of asthma. Innate immune responses are often mediated by receptors on mononuclear cells whose primary ligands arise from microorganisms. Many pathogens, especially viruses, target epithelial cells and affect the host immune response to those pathogens. The acquired immune response to an allergen is influenced by the nature of the innate immune system. Products of innate immune responses to microbes promote T_H_1-acquired responses. In the absence of T_H_1 responses, T_H_2 responses can dominate. Central to T_H_1/T_H_2 balance is the composition of contaminants that derive from microbes. In this review we examine the biology of the response to allergens, viruses, and bacterial products in the context of the development of allergy and asthma.

Allergy is a T_H_2-mediated immunologic phenomenon that is the most significant risk factor for development of childhood asthma. In the airway, the innate immune response to environmental agents gives rise to inflammation, enhancement of antigen presentation, and development of the primary (acquired) immune response. The inflammatory response results from the coordinated action of monocytes and macrophages, but it also involves responses of other cell types such as epithelial cells and neurons. Thus, while the immune response is central to the development of allergy, nonimmune structures also participate in this complex process.

A number of environmental factors have been reported to affect the development and severity of asthma, including outdoor air pollutants (e.g., particulates, ozone), indoor irritants, and agents such as environmental tobacco smoke. However, it can be argued that the most significant inhaled agents that modulate the development of respiratory allergy and asthma are biologics. Indeed, one important aspect of innate immunity involves the response of monocytes and macrophages, which is mediated by receptors whose primary ligands arise from various microorganisms. Furthermore, many pathogens, especially viruses, target epithelial cells, and the resulting responses of epithelial cells and surrounding monocytes greatly affect the host response to those pathogens.

It has been suggested that the primary acquired immune response to a given antigen is influenced by the nature of the innate immune system (and its associated cytokine response). Thus, products of innate responses to microbes that are more effectively cleared by IgG and T_H_1 inflammation might be expected to promote T_H_1-acquired responses. In the absence of such inflammation, T_H_2 responses can dominate, especially if inhaled bioaerosols contain agents that derive from multicellular organisms (which may mimic parasites). Ultimately, it is the total exposure and immune experience of an individual, coupled with genetic factors that control their innate and acquired immune responses, that determine if allergy develops in the airway. Central to T_H_1/T_H_2 balance is the composition of contaminants that derive from microbes. Hence, in this review we examine the biology of response to allergens, viruses, and bacterial products (primarily endotoxin) in the context of development of allergy and asthma.

## Cockroach, Dust Mite, Mold, Rodent, and Pet Allergens and the Induction of Asthma

The question of asthma induction usually brings to mind infants who experience asthma for the first time; however, at least two other examples illustrate the importance of allergen exposure to asthma incidence in adults. The first example is occupational asthma, especially that caused by laboratory animal allergy, where 25–30% of workers who are sensitized to laboratory animal allergens develop symptoms within 1 year of beginning work (Bush et al. 1998). About 25% of symptomatic workers have asthma symptoms, thereby making laboratory animal allergens a relatively common cause of incident asthma associated with a new allergen exposure in adults (Bush et al. 1998). A second example is the report of markedly increased rates of asthma in primitive villagers from the New Guinea highlands. In the 1980s adult men in these villages developed severe asthma, and 91% were sensitive to many allergens, including house dust mites (Dowse et al. 1985). Cotton blankets that had been donated by Western charities were found to be heavily contaminated with dust mites, thus suggesting that they had been presented with a new, unique exposure that led to sensitization and incident asthma (Dowse et al. 1985).

Children who develop asthma typically have symptoms by the age of 4–5 years, and a significant portion of them develop persistent asthma (Stein and Martinez 2004). Data from birth cohort studies suggest that atopy (defined by family history, other allergic manifestations such as eczema, elevated IgE, or sensitization) is a major risk factor for the development of childhood asthma (Lowe et al. 2002; Martinez et al. 1995; Platts-Mills et al. 1997; Wahn et al. 1997). In asthmatic children age 6 years and older, sensitization to airborne environmental allergens is very common (80–90% of cases), and the combination of sensitization and exposure is strongly associated with more severe disease (Rosenstreich et al. 1997).

### Allergens and their sources.

A number of allergen sources have been identified in the indoor environment (Table 1). House dust mites thrive in humid environments and live on human skin scales. Fecal particles, which contain the allergens, do not remain airborne for more than a few minutes after disturbance. Thus, the source has limited mobility, and exposure is limited primarily to bedding, carpeting, and upholstered furniture (Arlian and Platts-Mills 2001). Cockroaches cluster in narrow hiding places, coming out only to forage for food and water. The particles that contain the allergen are generally large, but the source is mobile so it is widespread in settled dust and, in many cases, accumulates in places inaccessible to cleaning (Eggleston and Arruda 2001). Rodents hide within walls and crevices, and leave high concentrations of allergen in inaccessible places. The allergens are found in urine and bodily secretions and are carried on small particles that remain airborne for extended periods of time. House dust is heavily contaminated, but removal is difficult because of the inaccessible reservoirs (Chapman and Wood 2001; Phipatanakul et al. 2004). Pets with fur produce allergens in their saliva and sebaceous secretions. Air sampling studies have shown that approximately 20–30% of airborne animal allergens are present on small particles of 1–5 μm diameter, in contrast to mite and cockroach allergens, which are carried on large particles of 10–40 μm diameter (Custovic et al. 1997; Luczynska et al. 1990). The animal allergens remain airborne for extended periods of time and are passively carried throughout the home as well as into public buildings and homes that have never housed a pet. After removal of a pet, household settled dust allergen levels decline over a period of 4–8 months (Wood et al. 1989). Air cleaners have been reported to reduce airborne pet allergen levels, but they have minimal effect on settled-dust allergen levels (Wood et al. 1998). The ecology of fungal allergen exposure is perhaps the least understood of all indoor allergens. Atopic persons are frequently sensitized, and fungi can easily be cultured from indoor dust and air. Fungal spores originate in the soil and are ubiquitous in the outdoor environment. The various fungal species and the levels of these spores fluctuate dramatically throughout the various seasons. These mold spores infiltrate the indoors via openings such as doors, windows, cracks and crevices. They are also transported inside by people and pets. Allergenic proteins have been isolated from fungi, but these allergens are not typically present in indoor environments. Recent data suggest that the allergens are only found in association with germinating fungal spores (Mitakakis et al. 2001).

### Exposure estimates.

In general, an exposure dose is determined by two factors: the exposure concentration (in the case of asthma, the airway or nasal concentration), and the exposure time. For allergens, the exposure concentration is uncertain. For simple sources, such as the house dust mite, allergen particles contaminate infested fabrics and then become airborne with disturbance (Platts-Mills and Chapman 1987). Particles are cleared by settling, but some are also absorbed onto walls, furniture, and other reservoirs (Platts-Mills and Chapman 1987). Reservoirs are in equilibrium with the air, regenerating airborne particles by physical disturbance or by air currents. Air concentrations are also influenced by ventilation and dilution by outside air. Finally, particles can be brought into the indoor environment by foot traffic or on clothing, generally adding to the reservoir dust and potentially adding to airborne particles that might be inhaled and contribute to an exposure dose.

Most studies of exposure have measured allergen levels in settled dust; only rarely have airborne concentrations been assessed. Settled dust and airborne dust mite allergen concentrations are highly variable, with reported coefficients of variation of 30% or more (Platts-Mills and Chapman 1987). Airborne concentrations of cat and other animal allergens are even more variable. Indeed, recent studies have shown that allergen concentrations in samples collected from the same home can vary by more than 3 orders of magnitude (Bollinger et al. 1996). This degree of uncertainty makes it difficult to determine the exposure dose that might be related to incident asthma. In general, airborne allergen concentrations do not correlate well with settled dust allergen concentrations (Swanson et al. 1989).

### Birth cohort studies of incident asthma.

Several birth cohort studies have reported a relationship between exposure and incident asthma. The Multicentre Allergy Study, a prospective study of 1,318 infants born in five German cities, was the first to describe the “allergic march” whereby children became sensitized first to food allergens (especially egg), then to inhalant allergens (such as dust mite and cat) up to 3 years later (Lau et al. 2000). Those who became allergic to foods were at greater risk for development of later sensitization to inhalant allergens. Incident sensitization was related in a dose-response fashion to dust mite and cat allergen exposure. Children who were sensitized to indoor allergens were at risk for incident asthma, but settled dust exposure doses were not directly related to incident asthma (Lau et al. 2000). In another prospective birth cohort study of 505 children in Boston, Massachusetts, exposure to cockroach allergen was found to be a risk factor for wheezing respiratory illness but not diagnosed asthma (Gold et al. 1999). This group also found that settled dust endotoxin concentrations were related to incident asthma (Park et al. 2001). In contrast, the Dutch PIAMA (Prevention and Incidence of Asthma and Mite Allergy) study found no relationship between settled dust exposures and incident asthma (Brunekreef et al. 2002).

### Preventing incident asthma.

To date, the results of two primary prevention trials have been reported. Arshad and Hide randomized a birth cohort of 124 mothers and their high risk infants to receive active or control environmental intervention. The active intervention included food avoidance measures during pregnancy and continued avoidance during breast-feeding. In addition, the child’s mattress was fitted with an allergen impervious cover. Asthma and sensitization were decreased in the first year of life in the active group, but the asthma effect was no longer statistically significant at 2, 4 and 8 years; however, a trend toward protection was consistent and was associated with *p*-values ranging from 0.10–0.06 (Arshad et al. 1992, 2003; Hide et al. 1994, 1996). A second intervention study was carried out in Manchester, United Kingdom, with 251 mothers and their newborn infants. The intervention included fitted mattress and pillow covers to the parent’s and child’s bed, laundry of bedding, and acaricide treatment of rugs and upholstered furniture. The intervention was successful in reducing mite allergen in the child’s bed and carpets by over 90% (Custovic et al. 2000). A recent article from this group reported significantly reduced airway resistance and a trend toward improved asthma symptoms in infants in the intervention group at 3 years of age (Woodcock et al. 2004).

## What Makes an Allergen an Allergen?

A number of epidemiologic studies carried out over the past 25 years have shown that IgE-mediated sensitization to indoor allergens (including those that derive from house dust mites, cats, dogs, rodents, cockroaches, and fungi) is a risk factor for the subsequent development of asthma (Platts-Mills et al. 1997). These studies include case–control studies, prospective studies, and allergen avoidance trials. Indeed, a recent longitudinal general population survey that followed over 600 children from the onset of asthma to age 26 years showed that sensitization to house dust mite was one of the strongest risk factors for persistence of asthma [odds ratio (OR) 2.41; 95% confidence interval (CI), 1.42–4.09] and also for predicting asthma relapses (OR 2.18; 95% CI, 1.18–4.00] (Sears et al. 2003).

Inhaled allergens are the most common cause of IgE responses worldwide. Allergens belong to distinct protein families with a diverse array of biologic functions. They include enzymes, ligand binding proteins (e.g., lipocalins), enzyme inhibitors, structural proteins, and regulatory proteins (Chapman et al. 2000). These proteins have been cloned, sequenced, and produced in high-level expression vectors. Purified recombinant allergens have immunoreactivity that is comparable to their natural counterparts, and they are being used to develop improved allergy diagnostics and vaccines. High-resolution crystal structures for the most important allergens are now available, including house dust mite (Der p 2), cat (Fel d 1), and cockroach (Bla g 2) allergens (Derewenda et al. 2002; Kaiser et al. 2003b; Pomes et al. 2002). More than 20 allergen structures have been resolved, and these molecules constitute the most well-defined groups of biomedically important proteins. Several databases have been developed for comparing the structure, biological function, and immunologic properties of allergens. A partial listing of available online databases is shown in Table 2.

### Why do allergens induce IgE responses?

**Figure 1:**
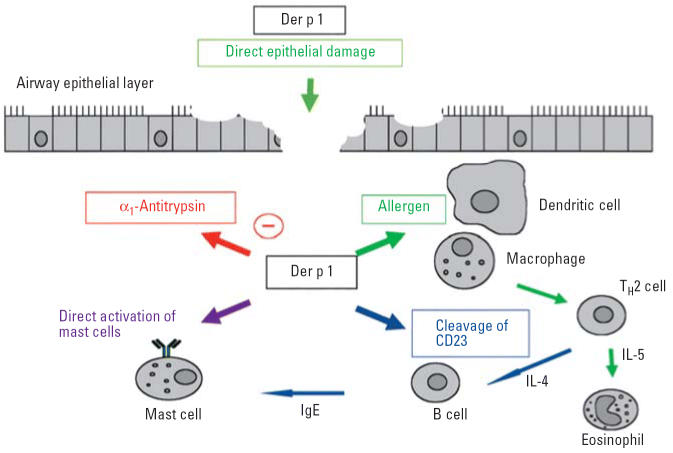
Biological effects of Der p 1. Reproduced with permission of Blackwell Publishing Ltd. (Sharma et al. 2003).

Two theories have been proposed to explain why allergens induce IgE responses (“allergenicity”). The “enzyme hypothesis” was originally developed as an explanation for why most dust mite allergens were proteolytic enzymes (principally cysteine and serine proteases, and chymotrypsin). Several lines of experimental evidence support this hypothesis (Figure 1) (Pomes et al. 2001; Sharma et al. 2003).

- Enzymatic activity directly promotes IgE synthesis through cleavage of the low-affinity IgE receptor (CD23) from activated B cells and by cleavage of the α subunit of the IL-2 receptor (CD25) on T cells (Hewitt et al. 1995; Shakib et al. 1998).
- Mite proteinases (Der p 1, Der p 3, Der p 6, and Der p 9) damage lung epithelium and increase bronchial permeability by inducing pulmonary epithelial cell detachment and disruption of intercellular tight junctions (Wan et al. 1999).
- Der p 1 induces production of proinflammatory cytokines *in vitro* [interleukin (IL)-8, IL-6, granulocyte-macrophage colony-stimulating factor] and induces IgE-independent mast cell and basophil degranulation (King et al. 1998).

This evidence also suggests that proteolytic allergens could contribute to lung damage and inflammation in asthma.

An alternative hypothesis is that the route of administration, dose of allergen inhaled (or ingested), and genetic predisposition are the principal factors that affect allergen recognition and development of allergen-specific T_H_2 responses that ultimately lead to IgE production. These factors apply to potent allergens, regardless of whether they are proteolytic enzymes. Recent structural studies have shown that several potent allergens are not enzymes. The group 2 mite allergens elicit IgE responses in 90% of mite allergic patients (Smith et al. 2001). The crystal structure of Der p 2 revealed a hydrophobic pocket within the molecule (Derewenda et al. 2002). Recent studies show that Der p 2 has structural homology to MD-2, a lipopolysaccharide (LPS) binding protein, and to a cholesterol binding protein C2 associated with Niemann-Pick disease (Gruber et al. 2004). The crystal structure of Fel d 1 revealed that the allergen was homologous to uteroglobin and contained an internal, asymmetric, amphipathic ligand binding pocket (Kaiser et al. 2003a, 2003b). Cockroach allergens are strongly associated with asthma among lower socioeconomic groups in innercity, rural, and suburban areas, yet none of the cockroach allergens identified to date has proteolytic activity. The most important allergen associated with IgE responses, Bla g 2, belongs to a subgroup of the aspartic proteinase family of enzymes that is enzymatically inactive (Arruda et al. 2001; Pomes et al. 2002). Attempts to render the Bla g 2 enzymatically active by selected site-directed mutagenesis of the active site catalytic triads have been largely unsuccessful. The high-resolution crystal structure of recombinant Bla g 2 defined the structural features that explain why the allergen is not an active enzyme and also showed that the allergen is a zinc binding protein (Pomes et al. 2002; Gustchina et al. 2005).

### Modified T_H_2 responses to allergens and immunological tolerance.

Dose-related effects of allergen exposure on IgE responses have been studied most extensively using cat allergen (Fel d 1). Several recent studies have reported that the prevalence of sensitization to cat is reduced when children live with one or more cats (Hesselmar et al. 1999). Moreover, exposure to high levels of Fel d 1 (> 20 μg/g dust) has been associated with a reduced prevalence of IgE antibody responses to Fel d 1 and an increase in IgG4 antibody responses (Custovic et al. 2001; Platts-Mills et al. 2001). At lower exposure levels (1–10 μg/g dust), the prevalence of IgE responses was increased. These studies have further demonstrated a “modified” T_H_2 response among a subset of individuals who develop IgG1 and IgG4 responses to Fel d 1, without an IgE response. These individuals appear to have a form of immunological tolerance to Fel d 1. In keeping with this, recent studies have identified tolerogenic T-cell peptides on Fel d 1 that are associated with the production of IL-10 *in vitro* and that stimulate increased IL-10 production in patients receiving allergen immunotherapy (Reefer et al. 2004). T-cell mapping experiments have identified peptides on Fel d 1 chain 1 that are associated with IL-5 production in allergic individuals and peptides associated with immune tolerance in modified T_H_2 responders (Platts-Mills et al. 2004; Reefer et al. 2004).

The induction of a form of immune tolerance following high-dose allergen exposure has obvious implications for the development of new vaccines to treat allergic diseases. New approaches to immunotherapy are being developed that rely on increasing the dose of allergen administered while reducing the potential for adverse reactions (Chapman et al. 2000). This effect has been achieved by generating genetically engineered “hypoallergens” that retain their ability to stimulate T cells but that have reduced IgE antibody binding capacity. Another approach has been the use of deoxycytidyl–deoxyguanosine dinucleotide (CpG)–coupled allergens, which demonstrate reduced allergenicity and promote the development of modified T_H_2 responses. An alternative strategy has been to use peptide-based vaccines to induce T-cell anergy or tolerance. Clinical trials are currently under way using hypoallergens, CpG-coupled allergens, and allergen peptides for immunotherapeutic purposes. Successful clinical outcomes have been reported in some of the initial trials using hypoallergens and CpG vaccines to treat pollen allergy (Chapman et al. 2000; Creticos et al. 2004; Niederberger et al. 2004). A recent study has also reported significant improvement in allergic symptoms using a vaccine containing several purified recombinant timothy pollen allergens (Jutel et al. 2005). It remains to be established whether any of these approaches will be effective for patients with asthma, who tend to be more difficult to treat with allergen immunotherapy. Nonetheless, these approaches offer the possibility of designing rational, safe, and more effective immunologic treatments for allergic disease.

## Viruses and Asthma

A number of studies have implicated viral lower respiratory tract infections early in life as a risk factor for the subsequent development of asthma (Piedimonte and Simoes 2002). In particular, it has been suggested that respiratory syncytial virus (RSV) infection may enhance the development of “allergic” inflammatory responses when the host is exposed to allergens after an episode of bronchiolitis.

Although RSV infection is usually self-limited and the virus is cleared from the respiratory tract of immune-competent children within several weeks, there is growing evidence to suggest that RSV infection may have long-term sequelae in the developing respiratory system (Piedimonte 2002). In fact, epidemiologic evidence from several retrospective studies as well as from more recent well-controlled prospective studies supports the association between early life RSV lower respiratory tract illness and recurrent episodes of wheezing and the development of asthma during the first decade of life (Sigurs et al. 2000; Stein et al. 1999). Indeed, RSV bronchiolitis and asthma share several clinical features (wheezing, increased work of breathing, tachypnea, and reversible changes in pulmonary function), but they also differ substantially in terms of response to bronchodilator and anti-inflammatory therapies. Despite extensive research, the precise molecular mechanisms and pathways by which RSV infection causes airway inflammation and affects long-term control of airway function subsequent to the initial insult remain unclear.

### Viral infection and neuroimmune interactions.

**Figure 2:**
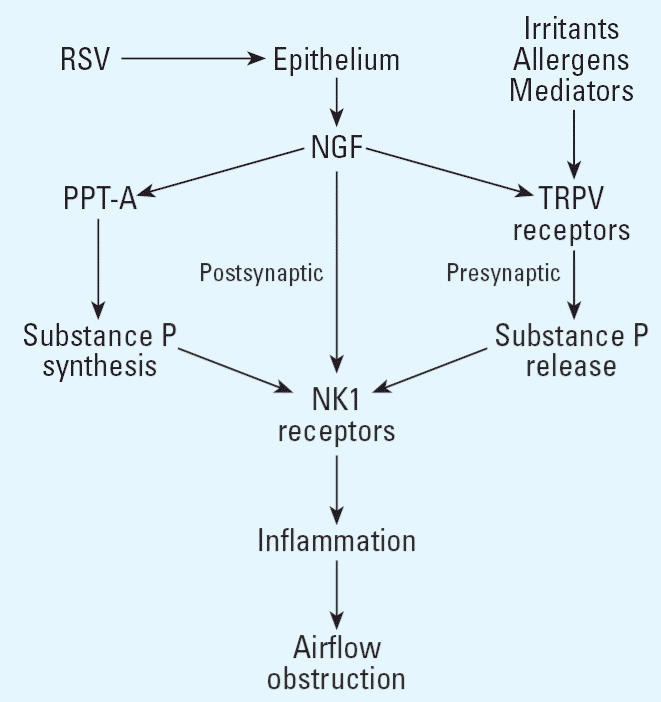
Viral infection and neuroimmune interactions. Abbreviations: RSV, respiratory syncytial virus; NGF, nerve growth factor; PPT-A, pre-protachykinin A; NK1, neurokinin 1; TRPV, transient receptor potential vanilloid.

Compromised epithelial integrity, the elaboration of local proinflammatory mediators, and dysfunction of neural pathways may influence airway responses to environmental stimuli. Some investigators postulate that infection with RSV or other viral pathogens can precipitate an imbalance in local cell-mediated immune responses (Lemanske 1998). Others hypothesize that infant bronchiolitis may result in alterations to neuronal pathways that influence airway smooth muscle tone and airway patency via the release of neurotransmitters (Larsen and Colasurdo 1999). Piedimonte has proposed that combined neuroimmune interactions primed by the virus can initiate and propagate a cascade of events leading to recurrent cycles of airway inflammation and obstruction (Figure 2) (Piedimonte 2001).

In the airway, a dense network of sensory nerve fibers is strategically placed just below the epithelial surface, so that any change in the bronchial environment may stimulate the release of the proinflammatory neuropeptide substance P (Piedimonte 1995). During RSV infection, stimulation of these nerves causes a marked increase in airway vascular permeability and results in an increase in overall inflammatory status (Piedimonte et al. 1999). Our work has revealed that these changes are mediated by the high affinity receptor for substance P (NK1 receptor), the expression of which is greatly increased by RSV (King et al. 2001; Piedimonte et al. 1999). This up-regulation presumably occurs at the pretranslational level because NK1 receptor mRNA levels increase substantially during RSV infection. We have also shown that T-lymphocyte subpopulations, predominantly CD4^+^ cells, within the bronchial-associated lymphoid tissue (BALT) of RSV-infected lungs express high levels of the NK1 receptor (Auais et al. 2003). As a consequence, stimulation of the sensory nerves by airborne irritants has the potential to cause a new inflammatory cycle that is mediated by the attraction of NK1 receptor–expressing T-helper lymphocytes and monocytes into the airway and activated by substance P. This mechanism may establish important neuroimmune interactions that undergo long-term dysregulation following RSV infection and predispose to airway inflammation and hyperreactivity.

### Viral infection, mast cells, and leukotrienes.

RSV also dramatically affects the distribution and function of mast cells in the airway mucosa (Wedde-Beer et al. 2002). Histopathological analysis with an antibody against tryptase identified numerous mast cells in sections from RSV-infected lungs, with an approximately 7-fold increase compared with the lungs of non-infected controls. In addition, most of these mast cells were in close spatial association with nerve fibers, suggesting functional mast cell–nerve interactions similar to those previously reported in other organ systems, particularly the skin, central nervous system, and gastrointestinal tract (Bauer and Razin 2000). Among the inflammatory mediators released from mast cells, cysteinyl leukotrienes (cysLTs) have been shown to cause airway inflammation and airway smooth muscle contraction during RSV infection, accounting for the wheezing observed in bronchiolitis. Increased leukotriene C_4_ (LTC_4_) levels were observed in nasopharyngeal secretions of children during the acute phase of RSV infection, and their concentration correlated with clinical severity, being higher in patients with lower respiratory tract involvement than in children with upper respiratory illness alone (van Schaik et al. 1999; Volovitz et al. 1988). Furthermore, cysLTs play critical roles in the pathophysiology of asthma and could represent an important component in the link between RSV and asthma.

Time course analysis of infected lung tissues indicated that the effect of RSV on 5-lipoxy-genase (5-LO) gene expression is transient; levels are maximal by 3 days postinoculation, already reduced by 5 days, and resolved by 30 days (Wedde-Beer et al. 2002). A similar profile was observed for the concentration of cysLTs in the same tissues, with almost complete return to pathogen-free levels by 5 days postinoculation. These findings suggest that the exaggerated neurogenic inflammation in the intrapulmonary airways infected by RSV in early life involves the concomitant release of cysLTs and activation of the cysLT1 receptor, as manifested by the potent inhibitory effect of the receptor antagonist montelukast on neurogenic-mediated vascular leakage.

On the basis of these studies, we speculate that following the early phase of the viral respiratory infection, leukotriene production and release rapidly return to baseline levels, but they can be reactivated by stimulation of the numerous mast cells still present in the lung tissues, for example, by substance P released upon stimulation of sensory nerve terminals. Another implication of these data is that the increased susceptibility of RSV-infected intra-pulmonary airways to the inflammatory effects of sensory nerves may be dependent, at least in part, on increased neurostimulation of mucosal mast cells, with consequent release of cysLTs. This effect, in turn, can amplify the release of tachykinins from sensory nerves, thereby forming a local neuron-mast cell feedback loop.

### Viral infection, nerve growth factor, and neurotrophins.

Recent studies show that RSV infection promotes a large increase in the expression of nerve growth factor (NGF) and neurotrophin receptors (Hu et al. 2002). NGF was the first discovered component of the neurotrophin family (Levi-Montalcini 1987), which includes the brain-derived neurotrophic factor (BDNF) and the neurotrophins 3 (NT-3) and 4/5 (NT-4/5). Neurotrophins modulate survival, differentiation and apoptosis of peripheral afferent and efferent neurons, and specifically control the expression of genes that encode the precursors of substance P and other peptide neurotransmitters. These effects are mediated by binding to high-affinity tyrosine kinase (*trk*) receptors (generally promoting neuron survival and differentiation) or to the low-affinity panneurotrophin receptor p75 (generally mediating apoptosis and death). The high-affinity receptor for NGF is the *trk*A subtype (Kernie and Parada 2000). Neurotrophins exert changes in the functional activity of peripheral neurons in a number of ways that collectively define “neuronal plasticity” (Renz 2001). Examples from studies *in vitro* and *in vivo* include increased production of neuro-transmitters, increased number of nerves that produce specific neuropeptides, and increased neurotransmitter release from nerve terminals mediated by increased expression and function of the vanilloid receptor TRPV1 (the capsaicin receptor). NGF is also synthesized in several nonneuronal cell types including epithelial and inflammatory cells (e.g., mast cells and CD4^+^ T cells) that also express *trk* receptors (Ehrhard et al. 1993; Leon et al. 1994; Nilsson et al. 1997). This function may target the innervation of specific tissues, but there is growing evidence that NGF functions as a potent and eclectic neuroimmunomodulator that releases and is released by a variety of inflammatory mediators. In particular, patients with bronchial asthma and allergic rhinoconjunctivitis display high serum levels of NGF, thereby suggesting an important pathogenetic role of neurotrophins in allergic disorders (Braun et al. 1999).

Because NGF is released from airway epithelial cells, increases the production and release of substance P and other tachykinins from adult sensory neurons, and induces sensory hyperinnervation in the airways of transgenic mice, it represents an ideal link between virus-infected respiratory epithelium and the dense subepithelial network of unmyelinated sensory fibers. RSV-induced release of NGF may lead to short- and long-term changes in the distribution and reactivity of sensory nerves across the respiratory tract, thus participating in exaggerated inflammatory reactions during and after the infection. NGF and its receptors may also amplify other immunologic and neuronal pathways contributing to airway inflammation and hyperreactivity. On the basis of these observations, we postulate that changes of neurotrophin expression in the respiratory tract may coordinate a variety of interactions between sensory afferent nerves and multiple components of the immune system and inflammatory pathways, thereby generating a pathophysiological link between early-life viral infections and childhood asthma.

## The Role of Endotoxin in Asthma

Allergens—such as those that derive from pollens, pets, rodents, cockroaches, house dust mites, or foods—might be considered harmless environmental antigens. Such antigens are recognized by the immune system, and the “normal” immune response is the development of clinical tolerance. In allergy and asthma, such antigens are recognized as “dangerous,” and the immune systems mounts an inflammatory response characterized by proliferation and activation of T_H_2 cells. Two key questions arise from this concept. First, how is the development of clinical tolerance regulated? Second, why is the immune system of atopic individuals not able to develop in this fashion?

### Role of early-life exposures and the hygiene hypothesis.

Increasing evidence suggests that prenatal and early postnatal environmental determinants play an important role in the development of allergy and asthma. Tolerance programming starts in early life, even before birth. Indeed, the presence of allergen-specific T cells has been demonstrated in humans at the time of birth, thus suggesting that specific immune responses can develop *in utero* (Prescott et al. 1999; Szepfalusi et al. 1997). Moreover, transplacental allergen transfer has been demonstrated in animals and humans (Holloway et al. 2000). Maturation of the fetal immune system occurs primarily during the first two trimesters of pregnancy. The development of clinical tolerance continues after birth and the first 2 years of life seems to be particularly important (Prescott et al. 1998; Prescott et al. 1999).

It is now well recognized that natural exposure to microbes through mucosal surfaces in the gastrointestinal tract, respiratory tract, and skin are critical for the development of clinical tolerance. These observations are directly linked to the “hygiene hypothesis,” which states that exposure to microbial antigens plays an important role in immunoprotection and is required for the development of clinical tolerance (Renz and Herz 2002). In fact, microbes are now viewed as important immunoregulators in addition to their role as pathogens. How are these facts linked to the development of allergy and asthma? Recent longitudinal and cross-sectional cohort studies have found that the traditional farming environment in the European Alps protects against the development of allergy and asthma (Braun-Fahrlander et al. 2002; von Mutius et al. 2000). Two factors were identified that presumably transmit this protection during the early postnatal period (the first year of life): consumption of raw (nonpasteurized) milk and daily exposure to farm animals (Braun-Fahrlander et al. 2002; von Mutius et al. 2000). To identify further the microbial components involved in this protection, investigators collected dust samples from over 800 families, and endotoxin (bacterial lipopolysaccharide or LPS) measurements were made. The results indicate a strong inverse association between natural, chronic exposure to endotoxin and the risk of allergic sensitization and clinical manifestations of respiratory tract allergy and asthma (Braun-Fahrlander et al. 2002).

### Endotoxin and the immune system.

**Figure 3:**
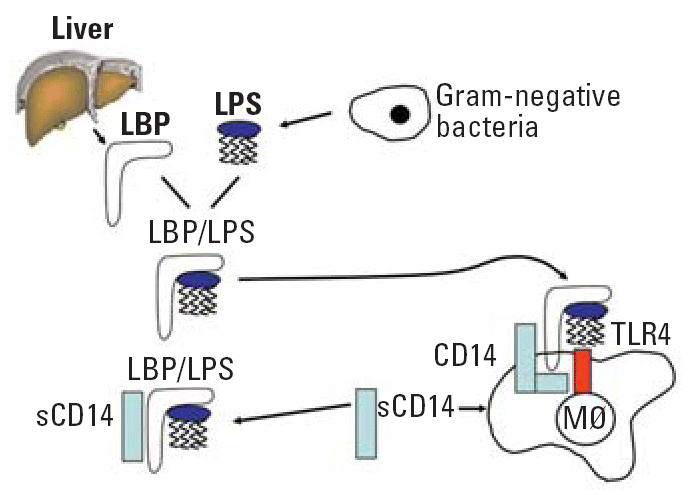
LPS recognition system. Abbreviation: LPS, lipopolysaccharide; LBP, lipopolysaccharide binding protein; CD, cluster of differentiation; sCD14, soluble CD14; TLR4, toll-like receptor 4; Mφ, macrophage.

The system of LPS recognition is highly complex and involves multiple components of the innate immune system. Recently, several molecules have been identified that play critical roles in this context. The LPS binding protein (LBP) acts as a carrier of LPS. This complex assembles with soluble or membrane bound CD14 molecules and allows recognition by the toll-like receptor 4 (TLR4) on the surface of immune cells such as macrophages. A schematic of this complex recognition system is illustrated in Figure 3.

To test further the concept that LPS exposure is linked to protection against the development of respiratory allergies, animal studies were conducted. Exposure of adult mice to LPS suppressed IgE production, airway inflammation, and development of bronchial hyperresponsiveness (Gerhold et al. 2003). LPS acted in a dose-dependent manner; high-dose exposure (equivalent to 100 μg LPS intranasally) promoted T_H_1 immune responses, and low-dose exposure (0.1 μg LPS intra-nasally) had a proallergic effect (Eisenbarth et al. 2002). To explore further the role of LPS in this process, a murine model of prenatal allergen exposure has been used. In this model LPS was administered intranasally to pregnant mice. Offspring were then sensitized to a conventional allergen (ovalbumin, OVA) followed by OVA aerosol challenges to induce experimental asthma. At birth, mice from LPS-exposed mothers had an elevated neonatal IFN-γ response. When these mice were sensitized to OVA, the development of anti-OVA IgE and IgG1 antibodies was markedly suppressed, whereas the levels of anti-OVA-IgG2a antibodies remained unchanged (Blumer et al. 2005). Furthermore, splenic mononuclear cells re-exposed *in vitro* to OVA produced significantly less IL-5 and IL-13 but not IFN-γ, thus indicating a selective suppression of the T_H_2 arm of the immune system. This effect was also reflected in the analysis of bronchoalveolar lavage fluid following OVA aerosol challenges. The influx of eosinophils, macrophages, and lymphocytes into the airways was also markedly suppressed; however, these mice remained hyperresponsive to metacholine. Together, these data provide experimental evidence that prenatal exposure to a microbial component such as LPS can modify the immune response to allergen exposure later in life. Further experiments are now under way to delineate the precise molecular mechanisms responsible for this effect.

### Other microbial components as immunomodulators.

Bacterial LPS is not the only microbial component that can act as an immunomodulator. In the studies cited above of European farmers, a polymorphism in the TLR-2 promoter has been associated with reduced allergic sensitization, asthma and hay fever (Eder et al. 2004). TLR-2 recognizes, among other things, peptidoglycans primarily produced by gram-positive bacteria, lipoprotein and zymosan, which is a component of yeast. Furthermore, the level of muramic acid, a major component of peptidoglycan that can be considered a marker for exposure to gram-positive bacteria, was inversely correlated with wheezing and asthma regardless of farming and endotoxin exposure (van Strien et al. 2004).

### An updated hygiene hypothesis.

Although it is clear that the prenatal and early postnatal environment influences the development of allergy and asthma, the exact nature of this influence is not completely understood. The updated “hygiene hypothesis” states that microbial load and chronic exposure to microbial compounds play an important role in the development of clinical tolerance and subsequently confer protection against allergic diseases. Future studies will be necessary to define precisely the components of this protective microbial load. Timing and duration of exposure seem to be critical. In terms of the duration, it is necessary to distinguish acute and chronic events. Dosing also seems to be critical, as experimental studies clearly indicate a differential effect of low- and high-dose exposures. Furthermore, the route of exposure must be considered. Nonmucosal LPS exposure is clearly an unwanted phenomenon that triggers an inflammatory response, whereas mucosal LPS exposure seems to be of particular benefit. Delineation of these and other aspects of the biology of microbes as immunomodulators might lead to the development of new avenues of allergy prevention and treatment in near future.

## Conclusion

In this article we have reviewed the role of allergens, viruses, and endotoxin in the development of allergy and asthma. While these agents may appear to be ubiquitous, there are variations in exposure to them that may affect the host. It seems likely that increasing endotoxin exposure and decreasing allergen and viral exposures would decrease development of allergic airway responses. The importance of these exposures cannot be overestimated, as they are sources of stimulatory ligands for lymphocytes and antigen-presenting cells. However, the complex immune and inflammatory interactions that result from exposure to these ligands are still not completely understood. As our understanding of the influence of these interactions on the development of allergy improves, novel interventions designed to modulate the host response to these asthmagenic exposures can be developed and implemented.

## Figures and Tables

**Table 1:** Indoor allergen sources.

Allergen	Animal source	Household source	Particle size (μm)	Distribution
Cockroach	Secretions	Mobile, hiding places	5–35	Dust, fabrics
Dust mite	Feces	Immobile, fastidious	5–35	Fabrics, beds
Rodent	Secretions, urine	Mobile, hiding places	1–15	Air, surfaces, fabrics
Pet	Secretions	Mobile, furniture	1–5	Air, widespread
Mold	NA	Moist surfaces, materials	5–10	Unknown

NA, not applicable.

**Table 2:** Online allergen databases.

Database	Reference
WHO/IUIS Allergen Nomenclature	IUIS 2004
Structural Database of Allergenic Proteins	UTMB 2004
Food Allergy Research and Resource Program	FARRP 2004
Protall	Protall 2004
ALLERbase	ALLERbase 2004
Allergome	Allergome 2004
Central Science Laboratory	CSL 2004

Abbreviations: IUIS, International Union of Immunological Societies; UTMB, University of Texas Medical Branch; WHO, World Health Organization.
